# Intensity modulated radiotherapy (IMRT) with concurrent chemotherapy as definitive treatment of locally advanced esophageal cancer

**DOI:** 10.1186/1748-717X-9-191

**Published:** 2014-08-29

**Authors:** Falk Roeder, Nils H Nicolay, Tam Nguyen, Ladan Saleh-Ebrahimi, Vasilis Askoxylakis, Tilman Bostel, Felix Zwicker, Juergen Debus, Carmen Timke, Peter E Huber

**Affiliations:** CCU Radiation Oncology, German Cancer Research Center (DKFZ), Heidelberg, Germany; Department of Radiation Oncology, Heidelberg University Hospital, Heidelberg, Germany; CCU Molecular and Radiation Oncology, German Cancer Research Center (DKFZ), Heidelberg, Germany; Department of Radiation Oncology, University of Munich (LMU), Munich, Germany; Department of Radiation Oncology, Massachusetts General Hospital, Boston, USA; Malteser Krankenhaus St. Franziskus-Hospital, Klinik fuer Strahlentherapie, Flensburg, Germany

**Keywords:** Esophageal cancer, IMRT, Concurrent chemoradiation

## Abstract

**Background:**

To report our experience with increased dose intensity-modulated radiation and concurrent systemic chemotherapy as definitive treatment of locally advanced esophageal cancer.

**Patients and methods:**

We analyzed 27 consecutive patients with histologically proven esophageal cancer, who were treated with increased-dose IMRT as part of their definitive therapy. The majority of patients had T3/4 and/or N1 disease (93%). Squamous cell carcinoma was the dominating histology (81%). IMRT was delivered in step-and-shoot technique in all patients using an integrated boost concept. The boost volume was covered with total doses of 56-60 Gy (single dose 2-2.14 Gy), while regional nodal regions received 50.4 Gy (single dose 1.8 Gy) in 28 fractions. Concurrent systemic therapy was scheduled in all patients and administered in 26 (96%). 17 patients received additional adjuvant systemic therapy. Loco-regional control, progression-free and overall survival as well as acute and late toxicities were retrospectively analyzed. In addition, quality of life was prospectively assessed according to the EORTC QLQs (QLQ-OG25, QLQ-H&N35 and QLQ-C30).

**Results:**

Radiotherapy was completed as planned in all but one patient (96%), and 21 patients received more than 80% of the planned concurrent systemic therapy. We observed ten locoregional failures, transferring into actuarial 1-, 2- and 3-year-locoregional control rates of 77%, 65% and 48%. Seven patients developed distant metastases, mainly to the lung (71%). The actuarial 1-, 2- and 3-year-disease free survival rates were 58%, 48% and 36%, and overall survival rates were 82%, 61% and 56%. The concept was well tolerated, both in the clinical objective examination and also according to the subjective answers to the QLQ questionnaire. 14 patients (52%) suffered from at least one acute CTC grade 3/4 toxicity, mostly hematological side effects or dysphagia. Severe late toxicities were reported in 6 patients (22%), mostly esophageal strictures and ulcerations. Severe side effects to skin, lung and heart were rare.

**Conclusion:**

IMRT with concurrent systemic therapy in the definitive treatment of esophageal cancer using an integrated boost concept with doses up to 60 Gy is feasible and yields good results with acceptable acute and late overall toxicity and low side effects to skin, lung and heart.

## Background

Esophageal cancer is the eighth most common cancer and the sixth leading reason for cancer-related mortality worldwide [1–4]. Despite therapeutic advances over last two decades, esophageal cancer still has a very poor prognosis, and the 5-year survival rates have been reported to be below 20% [4, 5].

Squamous cell carcinomas make up the majority of esophageal cancers worldwide with a very high incidence in the Middle East and Southern Asia [6, 7]. In recent years, the incidence of esophageal adenocarcinomas has increased considerably in Australia, Western Europe and the United States [1, 8, 9].

Surgical resection has been established as the main treatment option for locally limited cancer stages, and several surgical approaches are available for treatment depending on tumor localization and extent [10, 11]. In loco-regionally advanced but resectable stages, neoadjuvant chemoradiation followed by surgery results in better outcomes compared to surgery alone. However, none of the randomized trials has shown superiority of the trimodal tatment compared to definitive radiochemotherapy considering the overall survival of partients [12, 13].

Further challenging the necessity for surgery is the finding that almost 50% of affected patients are not amendable at all to major surgery for technical, functional or medical reasons at the time of diagnosis [14]. In those patients, definitive chemo-radiation therapy is the established treatment of choice. Early trials have shown beneficial effects compared to radiotherapy alone [15, 16], and definite chemo-radiotherapy has been shown in smaller studies to be comparable to surgery in patients with non-metastatic disease [17, 18]. Chemotherapeutic drugs cisplatin and 5-fluorouracil have been most commonly used in studies examining the effects of definitive chemo-radiotherapy in esophageal cancer [1], while the addition of targeted agent cetuximab to the chemo-radiation regime has shown an adverse outcome and increase in treatment-related toxicities compared to chemo-radiotherapy alone [19].

Dose considerations for esophageal radiotherapy have been based mainly on the results of the RTOG 94–05 trial, in which radiation doses of 50.4 Gy and 64.8 Gy were compared using non-intensity modulated radiation therapy [20, 21]. Somewhat surprisingly this trial did not show a difference in either loco-regional control or other endpoints including quality of life. Therefore the lower dose of 50.4 Gy has been established as the standard dose for esophageal chemo-radiotherapy. However, the still high rate of loco-regional failures indicates the need for further improvement of the local therapy component. Because the radiation techniques have considerably improved with the introduction of IMRT, we hypothesized that higher radiation doses delivered with IMRT could improve the clinical outcome. Thus we started to treat patients with locally advanced esophageal cancer who were candidates for definitive combined radio chemotherapy with increased dose intensity-modulated radiotherapy using an integrated boost concept.

## Patients and methods

### Patient characteristics

27 consecutive patients were treated with increased dose intensity-modulated radiation therapy as part of definitive treatment of esophageal cancer at the German Cancer Research Center (DKFZ) between 2005 and 2009, and were included in this analysis. Median age was 63 years (range 42 – 79 years) and 81% of patients were male (22 patients). Histological confirmation of esophageal cancer was obtained prior to treatment for all patients included in this analysis, and squamous cell carcinoma was the dominant histology (81%). 93% of patients suffered from T3 or T4 tumors and/or N1 disease. Detailed patient characteristics are listed in Table  1.

**Table 1:** **Patient characteristics**

Patient characteristics	n	%		n	%
**Age**			**Histology**		
median	63		squamous cell	22	81
min	42		adeno	3	11
max	79		other	2	7
**Gender**			**cN stage**		
male	22	81	0	7	26
female	5	19	1	20	74
**Localisation**			**cT stage**		
cervical	4	15	1	1	4
upper thoracic	7	26	2	3	11
central thoracic	10	37	3	21	78
lower thoracic	6	22	4	2	7
**Dose to PTV1 [Gy]**			**Dose to PTV2 [Gy]**		
median	56		median	50.4	
min	19.2		min	14.4	
max	62		max	52.2	
≥ 56 Gy	26	96	≥ 50.4 Gy	24	89
**simultaneous CHT**			**adjuvant CHT**		
yes	26	96	yes	17	63
no	1	4	no	10	37

n: number of patients,%: percentage, PTV: planning target volume; Gy: Gray, min: minimum; max: maximum, CHT: chemotherapy, cN: clinical N stage, cT: clinical T stage.

### Treatment application

**Figure 1:**
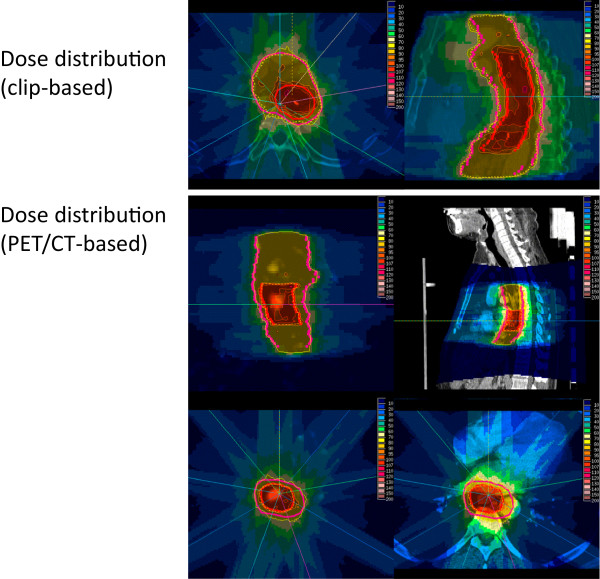
**Example pictures for dose distributions.** Sample pictures showing dose distributions as obtained from clip-based (upper panel) or PET/CT-based treatment planning. Red lines define boost volumes and pink lines planning target volumes.

Initial workup included at least clinical and laboratory examinations, endoscopy with biopsy, computed tomography of the primary tumor region and regional lymph nodes and abdominal ultrasound. The patients were staged according to the 6^th^ edition of the UICC TNM classification. In general, the GTV consisted of the primary tumor and involved lymph nodes as visible on contrast-enhanced CT. Primary tumor volume delineation was further supported by clips or FDG-PET information (Figure 1). Nodal GTV delineation was supported by available FDG-PET information. A margin of 1-2 cm was added in axial and 2–3 cm in longitudinal direction to the primary tumor to directly construct the boost volume (PTV1). The nodal volume (CTV2) included the regional lymph node regions within 4-5 cm in longitudinal direction from the edge of the primary tumor or at least 2-3 cm from the edge of the last involved lymph node. A margin of 0.5 cm was added to construct the PTV2. Margins could be reduced near critical organs at the discretion of the treating radiation oncologists.

Treatment delivery was performed by intensity-modulated radiation therapy (IMRT) using the step-and-shoot technique in all patients. Inverse treatment-planning was performed after immobilization of the patients in a vacuum pillow using the KonRad and VIRTOUS softwares developed at the German Cancer Research Center (DKFZ). Regional lymph node areas were irradiated to a median dose of 50.4 Gy in 28 fractions (range 14.4 to 52.2 Gy, single dose 1.8 Gy). The boost volume was covered with a median dose of 56 Gy (range 19.2 to 61.2 Gy). In 21 patients (78%) an integrated boost concept with slightly increased single doses of 2 to 2.14 Gy up to total doses of 56-60 Gy was used, while the remaining 6 patients received a sequential boost (range 9-10.8 Gy). 26 of the 27 patients completed radiation therapy with total doses of ≥ 56 Gy to the boost area. During treatment, image guidance with a CT on rails was performed at least once per week.

All patients were scheduled for concomitant systemic therapy. 26 of the 27 patients (96%) received concurrent systemic therapy, usually consisting of two courses cisplatin (20 mg/m^2^ BSA) and 5-fluorouracil (1000 mg/m^2^ BSA) on days 1 to 5 and days 21 to 25. Additionally, 17 patients (63%) also received adjuvant platinum-based chemotherapy.

### Clinical follow-up examinations and quality of life questionnaire

Regular follow up visits at our institution or the referring center included at least clinical examination, endoscopy and CT of the primary tumor and regional nodal regions. In case of clinical evidence for loco-regional recurrence or distant spread, additional tests or imaging modalities were performed to confirm or exclude disease progression at the discretion of the treating physician. Toxicity was scored according to CTCAE V3.0. In case of missing follow-up examinations, data was completed by calling the patient or the treating physician. Time to event data was calculated from the first day of radiation treatment until the last follow up information or until death. Loco-regional control was defined as absence of disease progression in the primary tumor region or regional lymph nodes. In patients without further assessment of loco-regional control, e.g. after development of distant spread, the date of the last information about the loco-regional status was used for calculation. Progression-free survival (PFS) was defined as absence of disease progression at any site or death of any cause. No subgroup analyses were performed due to the limited number of patients and events.

To better assess the quality of life of the patients undergoing combined radiochemotherapy we asked the patients to answer the EORTC questionnaire for the combined assessment of the quality of life (HRQL) for oesophageal cancer (QLQ-OG25) and specific questions from QLQ-H&N35 suitable for cervical or higher oesophageal cancer along with questions from QLQ-C30 addressing general cancer and therapy issues. QLQ-OG25 has six scales, dysphagia, eating restrictions, reflux, odynophagia, pain and anxiety.

The study is in compliance with the Declaration of Helsinki (Sixth Revision, 2008). Furthermore the study was approved by the Independent Ethics Committee of the Medical Faculty of the University of Heidelberg, Heidelberg, Germany (Ref. Nr.: S-490/2010).

### Statistics

Actuarial survival and loco-regional control were calculated using the Kaplan-Meier method and the QLQ questionnaire was analyzed using descriptive statistics, both using the Statistica software package (Statsoft 6.0).

## Results

### Local control and survival

**Figure 2:**
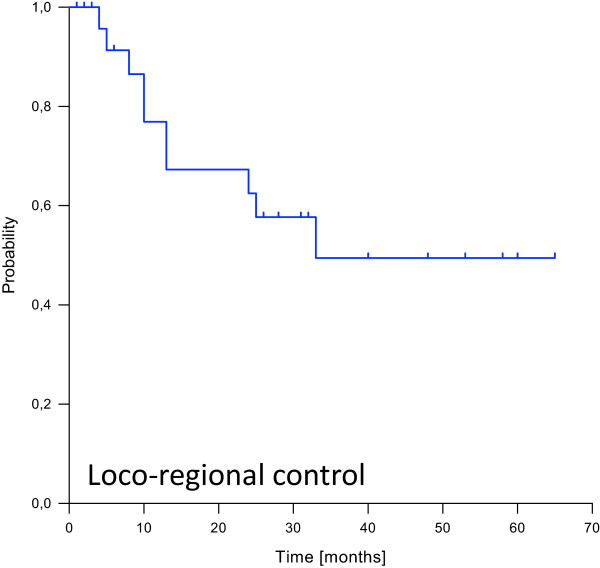
**Kaplan-Meier curve for loco-regional control probability.**

**Figure 3:**
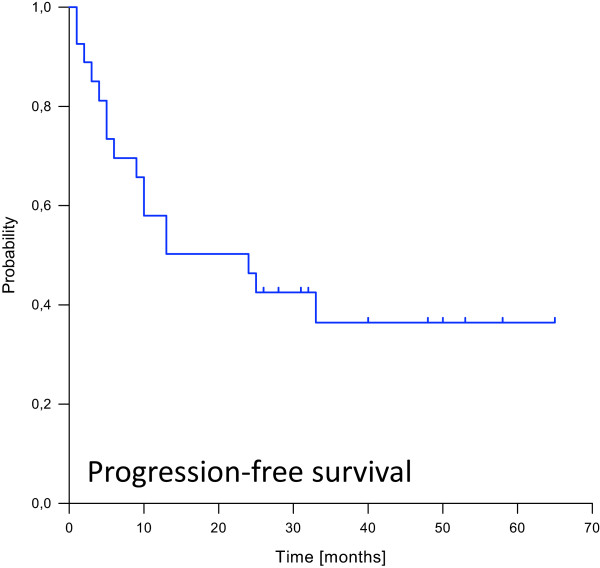
**Kaplan-Meier curve depicting progression-free survival probability.**

**Figure 4:**
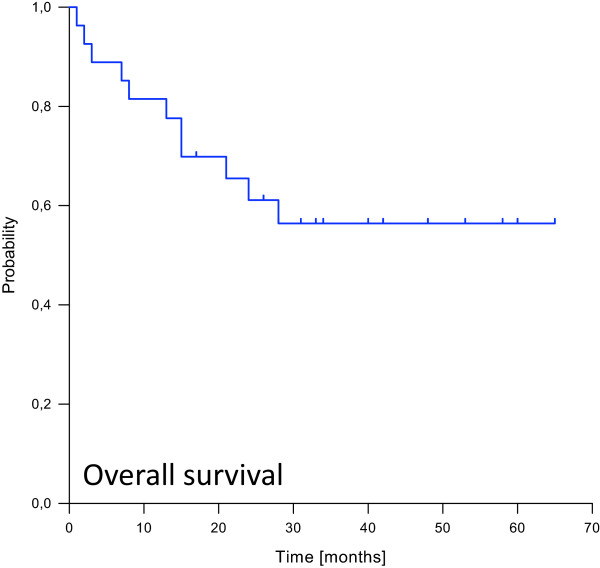
**Kaplan-Meier curve for overall survival probability.**

Median follow-up was 26 months (range 1 – 65 months) for the entire cohort and 34 months for surviving patients. Loco-regional recurrence was observed in 10 patients after a median time of 12 months, translating into estimated 1-, 2- and 3-year loco-regional control rates of 77%, 65% and 48% (Figure 2). 7 patients developed distant failures with a median time to occurrence of 8 months. 5 of them (71%) were located in the lung, while 2 (29%) patients suffered from liver metastases. The resulting estimated 1-, 2- and 3-year progression-free survival rates were 58%, 48% and 36% (Figure 3). Median overall survival was not reached. Estimated overall survival rates at 1, 2 and 3, years were 82%, 61% and 56% (Figure 4).

### Toxicity

Overall tolerance of definitive chemo-radiation was high. Radiotherapy was applied up to the planned dose in all except one patient (96%). 26 patients (96%) started concomitant systemic therapy and 21 patients (81%) received ≥ 80% of the scheduled cycles.

Acute side effects ≥ grade 3 occurred in 14 patients (52%). Hematological toxicities were most commonly observed with 4 patients developing grade 3 and 3 patients developing grade 4 leukopenia, 2 patients showing grade 4 thrombocytopenia and 1 patient suffering from grade 3 anemia. Therapy-related grade 3 or 4 dysphagia requiring PEG tube feeding was observed in 5 patients (19%). Acute skin and mucosal toxicities were uncommon with 1 patient developing an acneiforme rash and 2 patients reporting painful oral ulcers during treatment.

Severe late toxicities were detected in 6 patients (22%). Dysphagia was most commonly observed with 5 patients (19%) suffering from grade 3/4 esophageal toxicity due to ulcerations or strictures. Symptoms subsided in all but one patient after treatment with proton pump inhibitors or repeated dilatation. One patient required tracheal stenting after treatment due to the development of a tracheo-esophageal fistula. Severe late radiation-associated toxicities affecting skin, lung and heart were rare, with one patient suffering from a myocardial infarction and one from symptomatic pneumonitis.

QLQ questionnaire information suitable for analysis was available in 16 patients. Surprisingly, the patients reported that general effects of the disease or therapy were subjectively absent in almost 2/3 (62%) of patients at 3 months after radiotherapy end, 22% reported that ,some effects were present’, 10% reported ,significant effects’ and only 5% estimated the symptoms or other problems as “very significant”.

With respect to dysphagia and food intake, at 3 months after the end of radiotherapy 13% of patients used a feeding tube, but all together only 20% reported ‘significant’ eating restrictions with solid food, while soft food and fluids could be swallowed well in general. 60% had no heart burn at all, while 40% had mild heart burn. Mild reflux was only present in ~15%. In the majority (>75%) of patients. body weight was stable or increasing. 80% of patients did not need any pain medication. With respect to a general health 57% reported ‘not at all’, 26% reported ‘mild’, 9% ‘significant’, and 9% very significant restrictions on the quality of life. The mean self-evaluated health condition was 4.7 (almost ‘good’) on a scale from 1 (very sick) to 7 (excellent).

### Discussion

In this study, we present encouraging survival and toxicity data of esophageal patients treated with definitive high-dose chemo-radiotherapy. In our cohort of 27 consecutively treated patients, estimated overall survival rates at 1, 2 and 3, years were 82%, 61% and 56%, respectively, comparing well with other recent studies. A recent meta-analysis including 6 major studies with a total of 929 patients reported 2-year overall survival rates between 35 and 58% using definitive chemo-radiotherapy [22]. Another trial investigating 287 patients showed 2-year survival rates of 29% for patients suffering from esophageal squamous cell carcinoma and 19% for those with adenocarcinoma [23].

For a long time, dose considerations for esophageal chemo-radiotherapy have been based on the findings of the RTOG 94–05 study that compared an established dose regime of 50.4 Gy with high-dose irradiation using 64.8 Gy [21, 24]. In this study based on conventional 2D radiotherapy planning, higher doses did not offer any benefit regarding loco-regional control or survival while being associated with a higher percentage of treatment-related mortality. Quality of life analysis of the RTOG 94–05 study did not show a significant difference between 50.4 Gy and 64.8 Gy at 8 and 12 months after treatment [20]. Based on these findings, the recommended standard dose for esophageal cancer is 50.4 Gy [1, 21]. In contrast to the results presented in the RTOG 94–05 study, a meta-analysis published by Geh et al. suggested a dose–response relationship between increased radiation doses and pathological complete remission, warranting dose escalation studies in esophageal cancer [25].

The advent of highly conformal radiotherapy techniques and especially IMRT has generally enabled physicians to apply higher tumor doses without a significant increase in the doses absorbed by the surrounding normal tissue. A planning study using IMRT in esophageal cancer patients showed that dose escalation from 45 to 54 Gy was possible without significantly increasing normal tissue doses [26]. Additional data suggest that rotational IMRT techniques are able to further decrease doses to organs at risk compared to standard step-and-shoot IMRT [27, 28]. Another planning study in esophageal cancer comparing 2D radiotherapy plans using 50.4 Gy and IMRT plans using 64.8 Gy demonstrated a significant reduction of normal tissue dosage in the high-dose IMRT plans despite a 14 Gy increase in the tumor dose [29].

Due to esophageal movement and the risk of microscopic spread around the macroscopic tumor, large safety margins are commonly used as part of the clinical (CTV) and planning (PTV) target volumes. The RTOG 94-05 study based radiotherapy planning on 2D radiographs and recommended safety margins up to 5 cm. The advent of modern imaging modalities in defining target volumes in esophageal cancer patients has been subject to intense research as it holds the potential of more closely delineating target structures and decreasing PTV margins. Based on endoscopic ultrasound and PET/CT, planning studies using esophageal cancer data recommended 3 cm margins [21, 30]. In our dataset, treatment planning was based on initial CT scans and either ^18^ F-fluorodeoxyglucose PET/CT or endoscopic clip markings of the macroscopic tumor borders. PET/CT has been investigated as a means of delineating esophageal tumors and detecting metastatic lymph nodes for incorporation into the radiation treatment volume [31–33]. In a study published by Muijs et al. beneficial effects of PET/CT-based radiotherapy planning were demonstrated regarding target dose coverage and normal tissue complication probabilities [34]. In another study by Gondi et al. the availability of PET/CT information during treatment planning resulted in decreased GTV volumes in 63% of patients [35]. It is therefore conceivable that PET/CT-based radiotherapy planning may have the potential of further reducing safety margins, enabling higher treatment doses to the tumor and less normal tissue toxicities. However, further studies establishing the role of PET/CT in defining esophageal treatment volumes are warranted. Similarly, clip markings of the upper and lower end of the macroscopic tumor have shown benefits regarding target volume definition and may aid in applying increased treatment doses [36].

Additionally, image guidance has been examined as a means of increasing treatment precision and hence allowing dose escalation. Han et al. demonstrated significant inter-fractional setup errors for esophageal radiotherapy without daily image guidance [37]. In this setting, cone beam CT scans have been shown to be superior to portal imaging [38]. All of our patients received regular CT-based image guidance and repositioning prior to radiotherapy.

Treatment-related acute and late toxicities in our cohort of esophageal cancer patients were moderate. While about 50% of patients were observed to suffer from at least one acute grade 3 or 4 side effect, less than one quarter developed severe late toxicities. Hematological toxicities and dysphagia were the most common severe side effects noted in our patient group. IMRT treatment of esophageal cancer has previously been shown to result in reduced salivary gland toxicity and dysphagia, but increased rates of in-field toxicity, especially esophageal strictures [39]. Emami et al. reported a calculated 5% risk of esophageal stricture or perforation when 60 Gy were applied to more than one third of the esophagus [40]. Correspondingly, esophageal strictures were the most common late side effects observed in our patient group treated with high dose IMRT and concerned almost 20% of patients. However, these side effects are well amenable to treatment [41], and we found them to subside in all but one patient. Dose escalation studies using higher radiation doses up to 72 Gy incorporating endoluminal brachytherapy have shown even higher percentages of local side effects like esophagitis, ulcerations and strictures [42]. Other studies have evaluated out-of-field toxicities, especially lung and heart toxicities after esophageal radiotherapy and demonstrated beneficial effects of IMRT compared to 3D conformal treatments [43, 44]. However, due to the increased number of beams used for IMRT, several studies have found higher levels of pulmonary low-dose areas receiving less than 7 Gy (reviewed in [45]). Furthermore, high rates of hematological toxicity after esophageal chemo-radiotherapy with doses exceeding 60 Gy have been reported, but effects have often been attributed to concurrent systemic treatment [46, 47].

Interestingly, the questionnaire self-evaluation of patients after 3 months after the end of radiotherapy based on EORTC QLQs (QLQ-OG25, QLQ-H&N35 and QLQ-C30) focusing on dysphagia, eating restrictions, reflux, pain and anxiety revealed very encouraging results [8]. The subjective self-evaluation showed that ~50% of patients had no disease related symptoms, 25% encountered only mild problems and only 10% had significant or very significant restrictions of the quality of life.

Our study has limitations such as the small patient number and short follow-up period. Nevertheless, we demonstrated that intensity-modulated radiotherapy using increased doses up to 60 Gy via an integrated boost is feasible for the definitive treatment of esophageal cancer and can safely be applied in combination with concurrent systemic therapy. Our data also demonstrate encouraging results in terms of local control and survival with low acute and late side effects along with high satisfaction rates in self-evaluations. Taken together, our data may add to the literature that challenges the necessity for surgery in patients with locally advanced esophageal cancer. Furthermore, prospective studies investigating new radiation strategies with chemotherapy in larger cohorts of esophageal cancer patients are warranted.
